# Hypoglycemia and decreased insulin requirement caused by malignant insulinoma in a type 1 diabetic patient: when the hoof beats are from a zebra, not a horse

**DOI:** 10.1002/ccr3.927

**Published:** 2017-04-06

**Authors:** Hilde K. Gjelberg, Dag Hoem, Caroline S. Verbeke, Johan Eide, John G. Cooper, Anders Molven

**Affiliations:** ^1^ Department of PathologyHaukeland University HospitalBergenNorway; ^2^ Department of Gastrointestinal SurgeryHaukeland University HospitalBergenNorway; ^3^ Institute of Clinical MedicineUniversity of OsloOsloNorway; ^4^ Department of PathologyOslo University Hospital, RikshospitaletOsloNorway; ^5^ Department of MedicineStavanger University HospitalStavangerNorway; ^6^ Gade Laboratory for PathologyDepartment of Clinical MedicineUniversity of BergenBergenNorway; ^7^ KG Jebsen Center for Diabetes ResearchFaculty of Medicine and DentistryUniversity of BergenBergenNorway

**Keywords:** Chromogranin, diabetes, hypoglycemia, insulin secretion, insulinoma

## Abstract

Insulinomas are uncommon tumors, and in patients with diabetes mellitus they are extremely rare. We describe a patient with type 1 diabetes who developed malignant insulinoma. When hypoglycemic episodes persist in a patient with diabetes and treatment‐induced and other causes of hypoglycemia have been ruled out, an insulin‐producing tumor should be considered.

## Introduction

Insulinomas are insulin‐producing neuroendocrine neoplasms. They comprise the most common functional neuroendocrine tumor of the pancreas despite being rare, with an estimated incidence of only 0.7–4 cases per 1 million persons per year 1. Most insulinomas occur sporadically, but up to 10% are associated with the multiple endocrine neoplasia type 1 (MEN1) syndrome 2. The tumor occurs at any age with a median age at diagnosis of around 50 years, and there is a slight female preponderance 2. Approximately 5–10% of insulinomas are malignant 3; these tumors tend to be larger and have a higher mitotic count. A diagnosis of malignancy is reached by identification of metastatic disease, usually in lymph nodes or the liver.

Clinically, insulinomas are characterized by Whipple's triad 4, that is, the following three criteria: (1) presence of central nervous system or vasomotor symptoms of hypoglycemia; (2) low laboratory‐measured concentration of plasma glucose at the time of symptoms; and (3) prompt resolution of symptoms when the plasma glucose level returns to the normal range. The symptoms may be provoked by a 72‐h monitored fast. Upon confirmation of hypoglycemia, further laboratory investigations are necessary to establish its etiology. Inappropriately elevated insulin levels (>3 mIU/L) at the time of hypoglycemia (<2.5 mmol/L), together with elevated C‐peptide (>0.2 nmol/L) and proinsulin (≥25% or ≥22 pmol/L) levels 5, and the absence of plasma sulfonylurea are consistent with endogenous hyperinsulinism. When the diagnosis is established, different pre‐ and/or intraoperatively imaging modalities are used to localize the insulin‐producing tumor, which typically is solitary, small, and intrapancreatic 6, 7. Surgical resection is the treatment of choice. When possible, enucleation or segmental resection is performed to preserve endocrine and exocrine pancreatic function. The mean cure rate has been reported to be 93% in a review of more than 6000 insulinoma cases 8. The clinical course of metastatic insulinoma is variable, 25–35% of patients surviving longer than 5 years 6.

The incidence of insulinoma in patients with diabetes is considered to be lower than in the general population 9, and reported cases have been almost exclusively in type 2 diabetes mellitus (T2DM). Furthermore, hypoglycemia due to treatment with a hypoglycemic agent is not uncommon among patients with diabetes, and the detection of an insulinoma therefore represents a diagnostic challenge. We describe the clinical history and management of a malignant insulinoma diagnosed in a patient who had suffered from type 1 diabetes mellitus (T1DM) for 20 years. The patient has previously been included in summary tables describing 16 insulinoma cases referred to Haukeland University Hospital between 1986 and 2006 10.

## Case Report

A 43‐year‐old woman was referred to our hospital with a three‐year history of decreasing insulin requirements and frequent hypoglycemic episodes. Diabetes mellitus had been diagnosed when the patient was 23 years old and, initially, she was treated by diet only. Islet antibodies and C‐peptide were not measured at the time of diagnosis. At age 24, after a weight loss of 14 kg accompanied by polydipsia and polyuria, a diagnosis of T1DM was established and insulin treatment started. The patient's body mass index (BMI) at that time was 17.6 (height 170 cm, weight 51 kg). In the following years, her diabetes was well controlled, and there were no microvascular complications. She was also treated for hypothyroidism and hypertension from age 38.

The patient's family history included a sister with primary hyperparathyroidism, another sister with breast cancer diagnosed at age 43, a father who had T2DM, hypothyroidism and cardiovascular disease, a mother with vitamin B12 deficiency, and a cousin with hypothyroidism and celiac disease. The patient had two children, and her son developed T1DM at the age of 20. No cases of MEN1 syndrome were known in the family.

**Figure 1:**
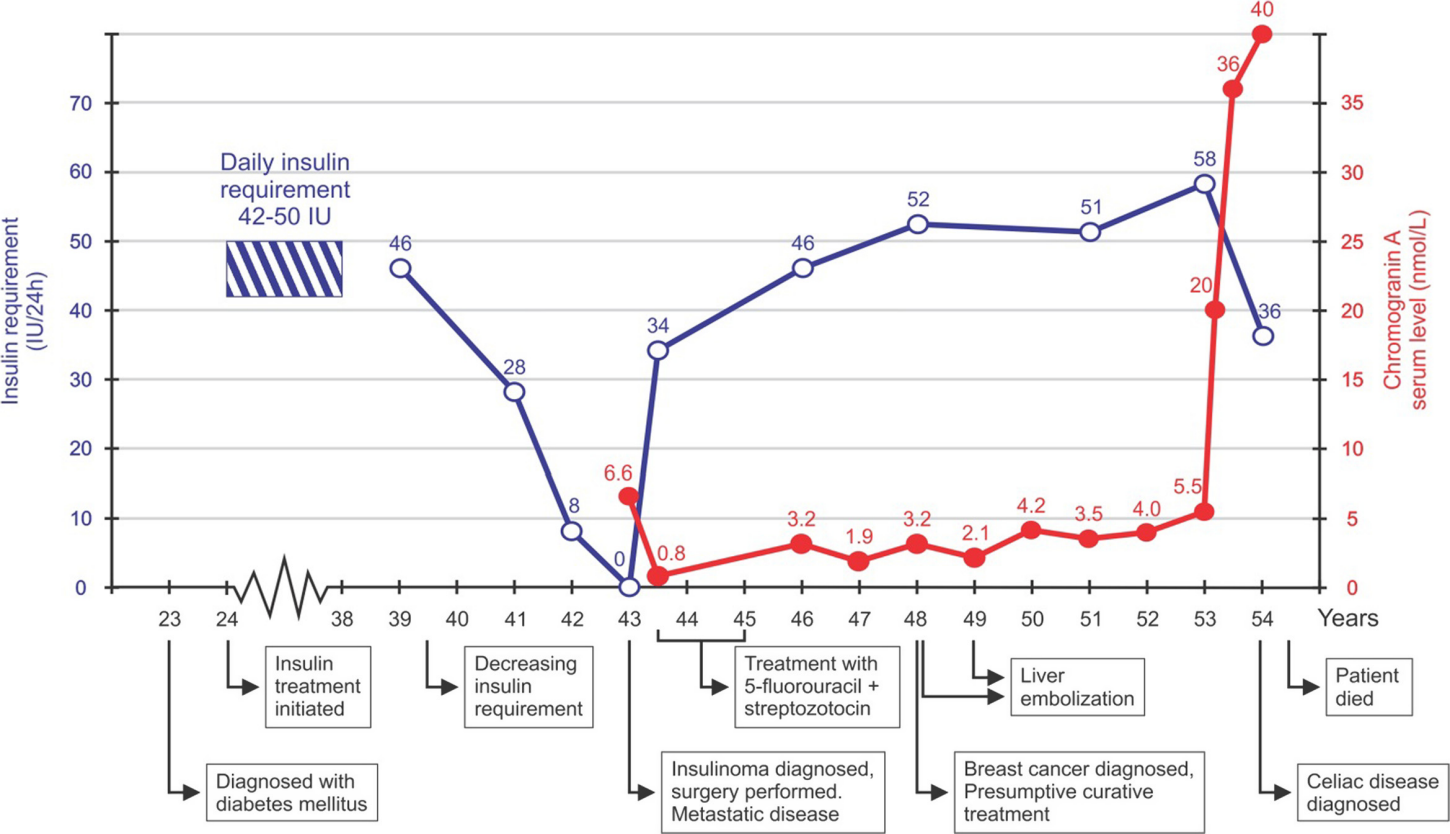
Time line illustrating major events in the patient's medical history, with age shown along the *x*‐axis. Insulin requirements and serum chromogranin A levels are indicated by open blue and solid red dots, respectively.

The patient was treated with an insulin pump during pregnancy when she was 31 years old. Otherwise her insulin requirements were stable, requiring approximately 42–50 international units (IU) of insulin daily, given as four bolus doses at mealtimes and one basal dose at bedtime. However, at age 40, her total daily insulin requirement started to decrease and was recorded as 28 IU at age 41 (Fig. 1). During the next year, intermediate‐acting (NPH) insulin was discontinued, and mealtime bolus doses were gradually reduced to a total of 8 IU per day. Despite these measures, the patient was experiencing frequent and long‐lasting hypoglycemic episodes, especially during the night. She also had detectable insulin C‐peptide level at 0.7 nmol/L (normal range, NR: 0.17–1.0 nmol/L). Chromogranin A was elevated at 6.6 nmol/L (NR: 0.4–2.0). However, the patient had been taking esomeprazole for abdominal discomfort, which may cause increased chromogranin A.

On admission at age 43, the patient reported a weight gain of 10 kg during the last 2 years, (current weight: 75 kg; BMI: 26.0). She looked fit and well. Her blood pressure was 140/80 mm Hg. There was no abdominal tenderness or palpable abdominal tumor. HbA1c was only slightly elevated at 7.0% (NR: 4.0–6.4%). Insulin treatment was discontinued, and the patient underwent a prolonged fast. After 24 h, she had symptoms of hypoglycemia, a bedside glucometer showed a blood glucose level of 1.9 mmol/L, and the fast was terminated. Laboratory‐measured plasma glucose was 2.8 mmol/L, C‐peptide was 0.58 nmol/L, and plasma insulin 3.8 mIU/L. The concurrent plasma insulin and glucose values were interpreted to be within normal limits, and therefore an insulinoma was considered to be less likely. A diagnosis of T2DM was also considered as the patient had been treated with diet only for the first year after her diabetes diagnosis, C‐peptide was measurable, and there was a family history of T2DM and cardiovascular disease. The patient was discharged without insulin treatment and advised to return to the hospital if hypoglycemia recurred.

Two months later, she was readmitted after a protracted hypoglycemic episode. Laboratory investigations revealed elevated glutamic acid decarboxylase (GAD) autoantibodies at 4 U/mL (NR: <1) and slightly elevated plasma glucagon at 84 pmol/L (NR: 7.2–72). The values for islet antigen‐2 autoantibodies, hemoglobin, erythrocyte sedimentation rate, C‐reactive protein, calcium, parathyroid hormone, prolactin, gastrin, human chorionic gonadotropin, alpha‐fetoprotein, pancreas polypeptide, adrenocorticotropic hormone, calcitonin, and liver tests were all within normal ranges. A new fast was then performed until hypoglycemia could be verified by the hospital laboratory. Sixteen hours after food intake, plasma glucose was 2.5 mmol/L, C‐peptide 0.56 nmol/L and plasma insulin 9.7 mIU/L. Two hours later, plasma glucose was 2.0 mmol/L, C‐peptide 0.60 nmol/L, and plasma insulin 8.2 mIU/L.

**Figure 2:**
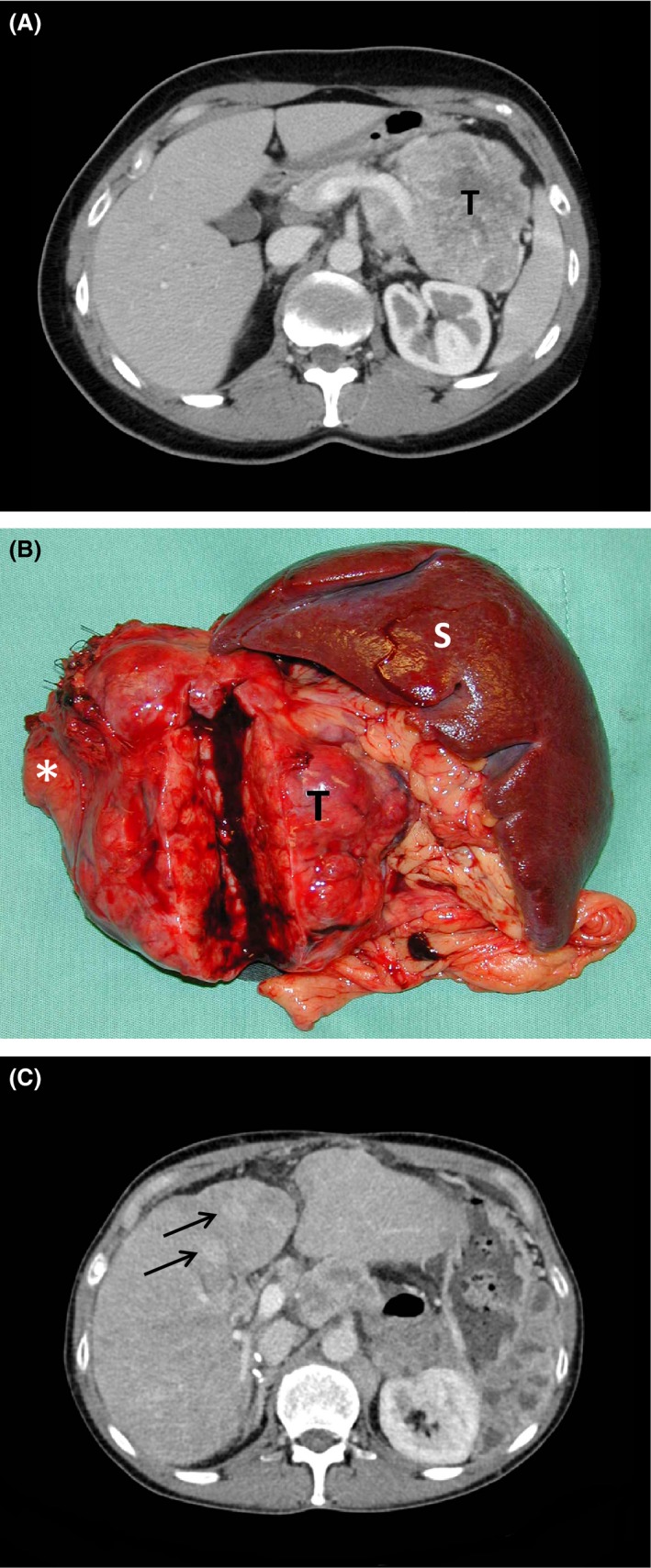
(A) Preoperative computed tomography image at the level of cauda pancreatis, portal venous contrast phase. A large tumor (T) is seen in the pancreatic tail. (B) The surgical specimen consisting of the pancreatic tail with a large, circumscribed tumor (T), the spleen (S), and hilar and peripancreatic fat. An incision has been made in the tumor to expose the inner surface. The asterisk indicates the resection margin toward the residual pancreas. (C) Similar image as A, 11 years after surgery. Two hypervascular liver metastases are evident (arrows).

Endogenous hyperinsulinism was now considered to be the cause of the patient's hypoglycemia. Abdominal ultrasound examination showed a hypoechoic lesion with a diameter of around 10 cm in the pancreatic tail. CT examination (Fig. 2A) confirmed the presence of a large retroperitoneal tumor and also revealed enlarged retroperitoneal lymph nodes as well as two possible liver metastases. Octreotide scintigraphy demonstrated increased uptake in the large tumor, but none in the liver or other organs. The patient underwent surgery, and the pancreatic lesion was removed. The surgical specimen (Fig. 2B) consisted of the body and tail of the pancreas with a well‐circumscribed, large central tumor measuring 11 × 7 × 5.5 cm, the spleen, hilar and peripancreatic fat, lymph nodes, and left adrenal. Also resected were two liver metastases, each measuring 1 × 0.5 × 0.5 cm. The tumor was partly demarcated by a thin pseudocapsule of connective tissue and showed a light gray‐yellowish cut surface with a variegated, mainly soft consistency.

**Figure 3:**
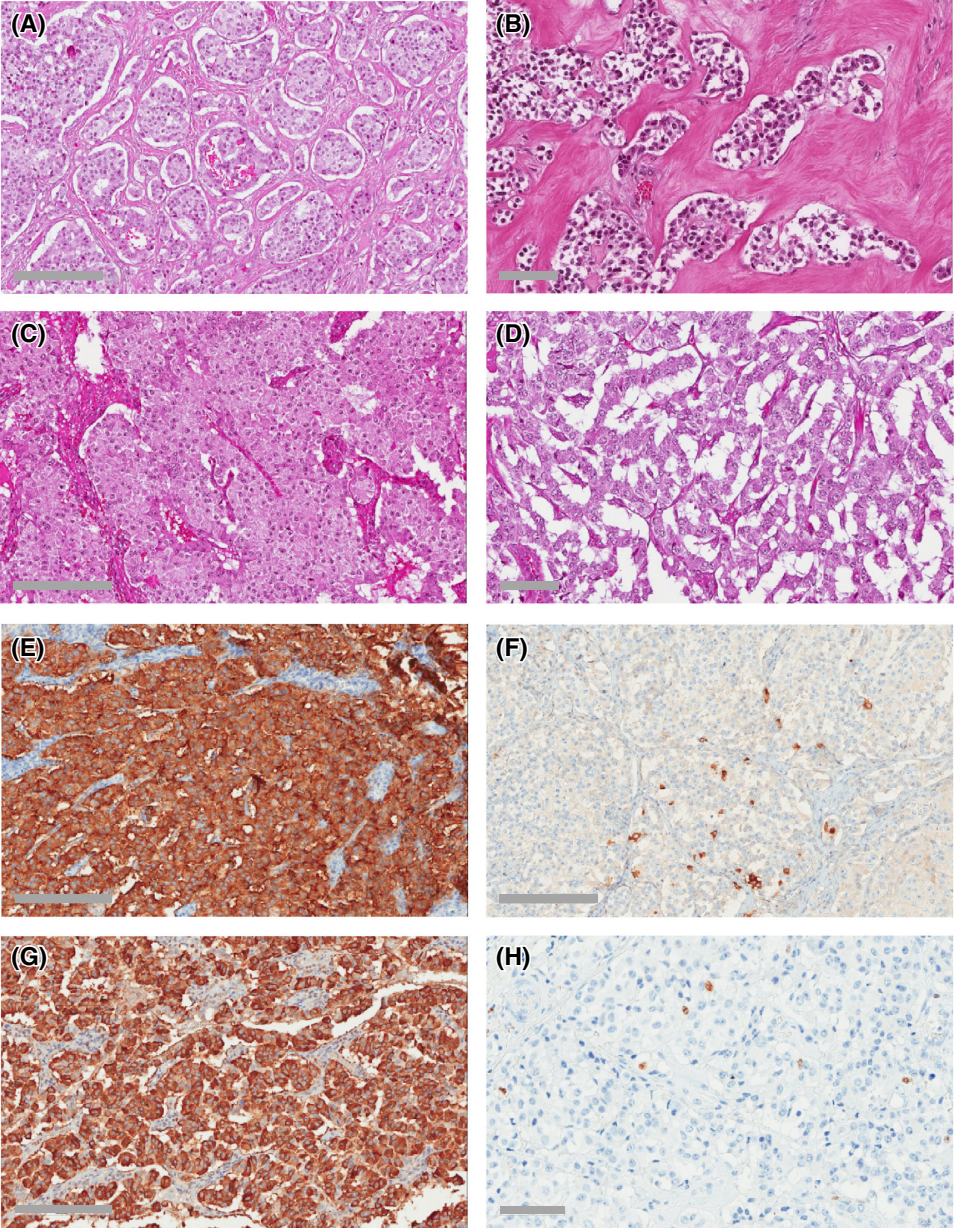
(A–D) Hematoxylin/eosin histological images of the tumor illustrating its varying growth patterns. (A and B) Nested growth pattern with various amounts of sclerotic stroma. (C) Solid diffuse growth pattern. (D) Trabecular pattern. (E–H) Immunohistological profile of the tumor tissue. (E) Synaptophysin. (F) Glucagon. (G) Insulin. (H) Proliferation marker Ki‐67. Scale bars: 200 *μ*m (A, C, E, F, G); 75 *μ*m (B, D, H).

Histological examination (Fig. 3A–D) revealed an epithelial tumor with varying histological growth pattern, including nested, trabecular, gyriform, and solid areas. There was a varying amount of fibrous stroma, focally with a marked sclerotic quality. The tumor cells showed a varying degree of slightly granular eosinophilic to amphophilic cytoplasm and moderately pleomorphic round to oval nuclei with coarsely clumped chromatin and one or more centrally placed distinct nucleoli. Mitoses were nearly undetectable; <1 mitosis per 10 high‐power fields on average. There was no inflammatory response.

**Figure 4:**
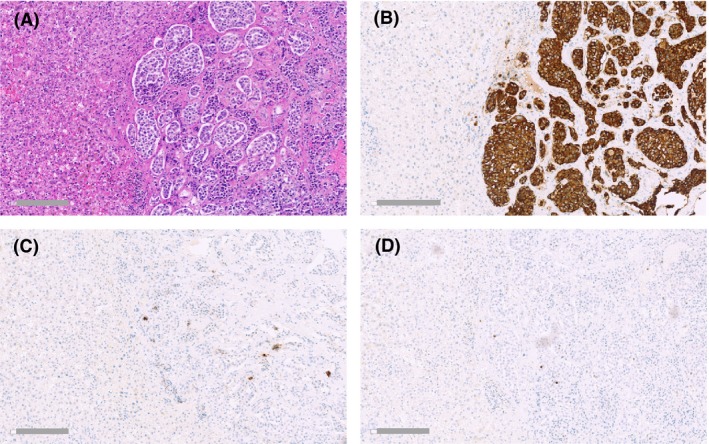
Liver tissue with metastatic tumor deposits (right part of image) resected during the surgical removal of the primary tumor. Consecutive sections. (A) Hematoxylin/eosin. (B) Synaptophysin. (C) Insulin. (D) Proliferation marker Ki‐67. Scale bars: 200 *μ*m.

Routine immunohistochemistry (Fig. 3E–H) showed diffuse and strong positivity for chromogranin A and synaptophysin. Insulin expression was diffuse and moderate, with smaller areas exhibiting strong positivity. Staining for somatostatin showed diffuse positivity with varying intensity. A minority of the tumor cells exhibited positivity for glucagon. Staining for vimentin, calcitonin, gastrin, human growth hormone, ACTH, and thyroid transcription factor was negative. Ki‐67 proliferation index was <1%. Two peripancreatic lymph node metastases and two liver metastases showed the same morphological pattern and immunohistochemistry profile as the primary tumor (Fig. 4), that is, diffuse and strong immunoreactivity for chromogranin A and synaptophysin, although only focal immunoreactivity for insulin. The left adrenal was unremarkable.

**Figure 5:**
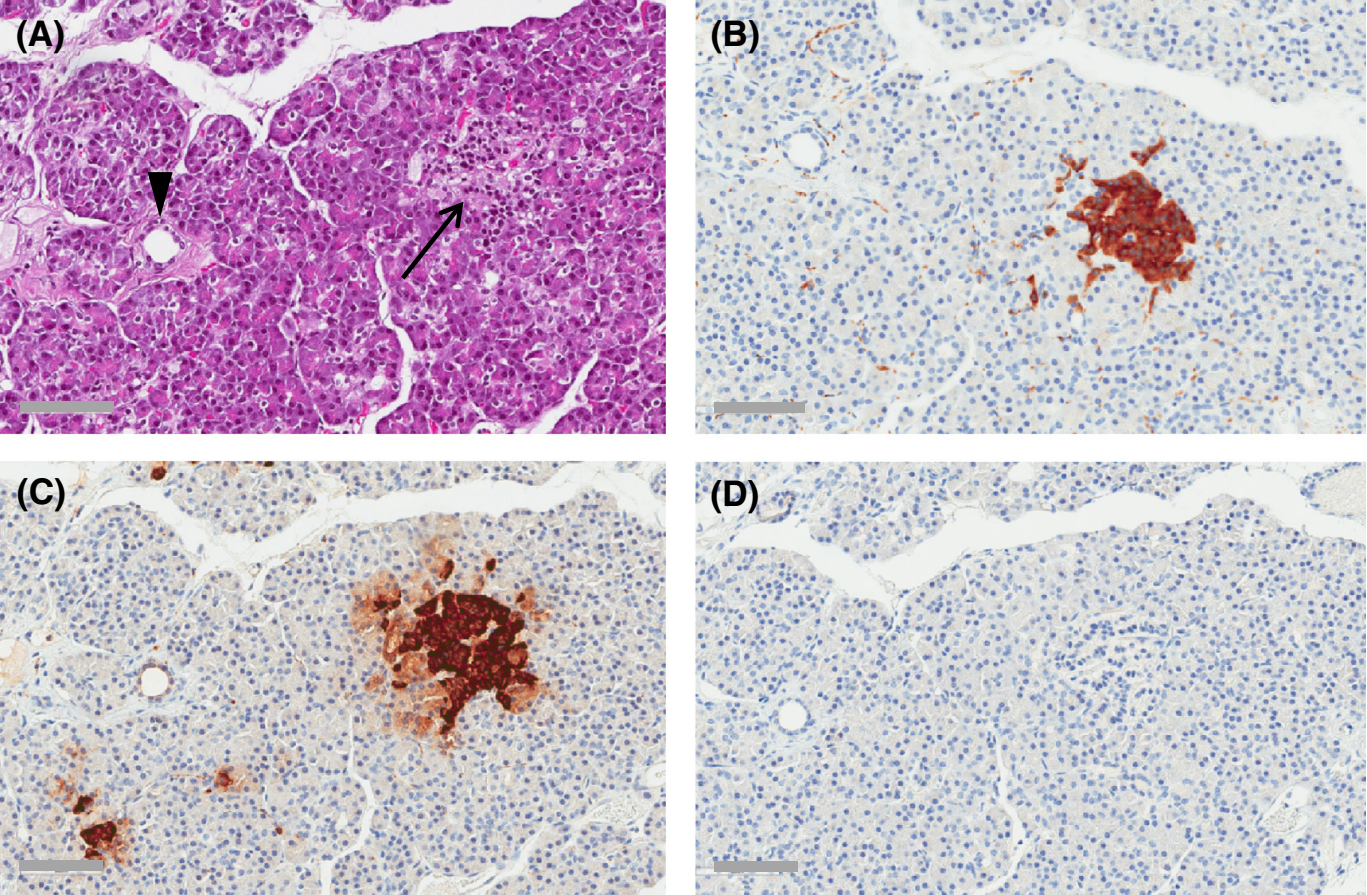
Nontumorous pancreatic tissue showing an islet of Langerhans (arrow) and a small pancreatic duct (arrowhead). Consecutive sections. (A) Hematoxylin/eosin. (B) An islet highlighted by immunohistochemical staining for synaptophysin. (C) Glucagon. (D) No immunoreactivity for insulin. Scale bars: 80 *μ*m.

Islets in the nontumorous pancreatic tissue of the resection specimen (Fig. 5) consisted almost exclusively of glucagon‐positive cells. There was no positivity for insulin. Neither the islets nor the exocrine parenchyma showed significant infiltration of inflammatory cells. Taken together, the clinical, morphological, and immunohistochemical findings were those of a malignant insulinoma.

Immediately after surgery, the plasma glucose concentration increased, C‐peptide became undetectable, and chromogranin A fell to 0.8 nmol/L (Fig. 1). Insulin therapy was reintroduced. The patient was discharged on a basal/bolus regime of 34 IU insulin daily. Chromogranin A and C‐peptide were undetectable, and it was decided not to give adjuvant treatment with a somatostatin analog (octreotide) or other chemotherapy as all known tumor tissue had been removed.

Five months after surgery, a CT examination revealed two new liver metastases, while C‐peptide and chromogranin A remained undetectable. Because the patient was asymptomatic, further treatment was not initiated at this point. Six months later, numerous liver and regional lymph node metastases had emerged, giving rise to pleiotropic symptoms, mainly weight loss and night sweats, but not those of hyperinsulinemia. Liver function tests were normal, and C‐peptide and chromogranin A were undetectable. Due to multifocal metastatic disease, the patient was not eligible for surgery and was treated palliatively with 5‐fluorouracil and streptozocin. Initially, there was remarkable alleviation of symptoms, and the patient's performance status was close to normal. Follow‐up CT scans showed regression of the largest liver metastasis, and there was no sign of disease progression for almost 2 years. She then developed icterus and a marked elevation of liver enzymes ascribed to chemotherapy‐induced hepatotoxicity. Streptozocin and 5‐fluorouracil were therefore discontinued.

Subsequently, liver enzymes normalized, but the size and number of liver metastases increased. There was reoccurrence of night sweats and slightly elevated chromogranin A (3.2 nmol/L). Hypoglycemic episodes were not recorded. A biopsy from one of the liver metastases showed strong immunoreactivity for synaptophysin and chromogranin A, but none for insulin. Attempted treatment with octreotide and interferon had to be terminated because of hepatotoxicity.

Four and half years after the initial surgery, the patient underwent whole‐body [^11^C]5‐hydroxytryptophan positron emission tomography at Uppsala University Hospital. There was uptake in the liver and in para‐aortic lymph nodes. Due to the risk of liver failure and the existence of extrahepatic disease, the patient was not considered to be a candidate for treatment with radioactive lutetium or liver transplantation. During the following months, further disease progression occurred with multiple liver metastases and ascites, and the patient was treated with liver embolization (transarterial embolization with microspheres) at Rikshospitalet, Oslo. In three subsequent sessions over a 1.5‐year period, a large part of the right liver lobe and segments 2, 3, and 4 of the left lobe were embolized. This procedure and spironolactone treatment alleviated symptoms for a period of time.

Five years after the insulinoma diagnosis, at age 48, the patient was diagnosed with lobular carcinoma of the right breast. She was treated with curative intent, and two foci (5 and 11 mm) of a grade 2 lobular carcinoma were completely resected. Estrogen and progesteron receptors were positive and HER2 negative. A sentinel node revealed one micrometastasis. The patient was given postoperative locoregional radiation therapy (2 Gy × 25) and antiestrogen treatment with tamoxifen. Follow‐up showed no breast cancer recurrence.

Seven years after pancreatic surgery, and following several months of stable disease, there was again progression of the residual liver metastases, and chromogranin A levels were rising. The patient was given second‐line treatment with temozolomide and capecitabine for nearly 2 years. During this time, she developed portal vein thrombosis, which was treated with injections of low‐molecular‐weight heparin. She did not have any hypoglycemic episodes, and her diabetes was well regulated with stable insulin requirements. She was then observed for 1 year without treatment.

Ten years postsurgery, temozolomide and capecitabine were reintroduced, but had to be discontinued due to intractable side effects (diarrhea, abdominal pain, general weakness). However, the patient's diarrhea and abdominal pain did not improve, and celiac disease was diagnosed based on elevated tissue transglutaminase‐immunoglobulin A, positive HLA DQ2/DQ8 and typical findings in duodenal biopsies. The abdominal symptoms improved with a gluten‐free diet. Abdominal CT showed slow progression of the liver metastases (Fig. 2C).

During this period, chromogranin A levels started to rise rapidly, and 11.5 years postsurgery, the patient was admitted to hospital with acute confusion and in poor general condition. Octreotide scintigraphy showed, in addition to multiple liver and lymph node metastases, uptake in the peritoneum, suspicious for peritoneal carcinomatosis. A CT scan of the head did not reveal any specific pathological findings. There were no hypoglycemic episodes. The patient died a few days later. A postmortem examination was not permitted by the relatives.

## Discussion

In diabetes mellitus, hypoglycemic episodes are relatively common and then usually associated with the use of exogenous insulin or insulin secretagogues. The co‐occurrence of diabetes and insulinoma poses diagnostic challenges and is sufficiently rare to deserve individual case reports. In a series of 313 confirmed cases of insulinoma at the Mayo Clinic from 1927 to 1992, there was only one case of a functioning insulinoma in a patient with established T2DM diagnosis 9. In 1996, a review of the literature retrieved 17 documented case reports of insulinomas in patients with T2DM 11. We have found only two published case reports of insulinoma in patients with a pre‐existing diagnosis of T1DM 11, 12, although it should be noted that tumor immunoreactivity to insulin was documented in only one of the patients 12. Moreover, two insulinoma patients have been diagnosed with T1DM soon after surgical resection of the tumor, including one case with documented insulitis and the absence of beta cells in the nontumorous pancreatic tissue 13.

For the patient presented here, the evidence for an autoimmune etiology of her diabetes is strong. A need for insulin treatment within 1 year of diagnosis in a nonobese young person is in itself suggestive of T1DM. Moreover, elevated GAD autoantibodies were detected, and there was no sign of insulin expression in the islets of the nontumorous pancreatic tissue removed during surgery. C‐peptide was undetectable postoperatively and insulin requirement re‐emerged immediately. Finally, the patient had two other autoimmune disorders (hypothyroidism, celiac disease) known to be associated with T1DM as well as a son with this disease.

The histological appearance of the tumor together with the immunoprofile (strong, diffuse positivity for chromogranin A and synaptophysin) confirmed the diagnosis of a neuroendocrine tumor. The focal distribution of tumor cells intensely positive for insulin together with clinical and laboratory signs of insulin production is diagnostic for an insulinoma 6. The presence of liver metastases and the course of the disease demonstrated the malignancy of the tumor.

In retrospect, the insulinoma should have been diagnosed during the patient's first hospital admission at age 43. The reason for the delayed diagnosis was partly a belief that insulinoma and T1DM could not co‐exist. Moreover, the fast was stopped too early (after 24 h) based on bedside blood glucose measurements that overestimated the degree of hypoglycemia. A prolonged fast (up to 72 h) has a sensitivity approaching 100% for detecting functioning insulinoma 14. In addition, the C‐peptide level at the end of the first fast was not assessed carefully enough. C‐peptide measurement can exclude exogenous insulin administration as cause of hyperinsulinism, and a concentration above 0.2 nmol/L in a patient with hypoglycemia is suggestive of insulinoma 5, 14.

Given that the prevalence of diabetes mellitus is 5–10% in industrial countries and that the incidence of insulinoma is 0.7–4 per 1,000,000 per year 1, co‐existence of the disorders should be more frequent than the literature indicates. It is possible that the presence of diabetes prevents the development of insulinoma or reduces the clinical symptoms. In addition, hypoglycemia in diabetic subjects is frequently observed and almost always caused by overtreatment with glucose‐lowering agents, making it easy to neglect alternative explanations for persistent hypoglycemia.

The demonstration of insulinoma in T1DM raises some interesting questions with regard to the underlying pathophysiology and origin of the tumor. The insulin‐producing malignant cells must have been able to avoid the autoimmune attack that otherwise had completely destroyed the beta cells of the patient. Notably, there was no evidence of an inflammatory response, neither in the resected tumor nor in the normal pancreatic tissue. Whether there was a lack of autoantigen expression in the insulinoma cells or they were able to escape immune surveillance in other ways will remain an open question. In this regard, it should be noted that although the large majority of insulinomas are benign 3, 8, the insulinomas of both the current and the two previously published T1DM patients were malignant 11, 12.

In some cases, such as in the MEN syndrome and in autoimmune chronic gastritis, neuroendocrine tumors may develop in the context of endocrine cell hyperplasia 15. In long‐standing T1DM, the pancreas has been depleted of normal beta cells (Fig. 5D). It is therefore difficult to envisage that the insulinoma of the present case developed from hyperplastic or normal beta cells. Instead, we propose that that the pluripotency of uncommitted stem cells is required to give rise to an insulin‐producing tumor in a patient who lacks normally functioning beta cells and where such cells are likely to be constantly attacked by the immune system.

## Conclusion

This case report and previous publications show that insulinoma can occur in individuals with diabetes, even in patients with long‐standing T1DM. Thus, when hypoglycemic episodes persist in a diabetic subject despite reduction or discontinuation of hypoglycemic treatment, and other causes of low blood glucose level have been ruled out, the presence of an insulin‐producing tumor should be considered.

## Authorship

HKG: evaluated the patient's medical records, did the literature review, wrote the article draft, and made the figures. DH: operated the patient, provided the patient's consent, and contributed to the writing process. CSV: followed up on all pathology data and made critical revisions of the text and figures. JE: did the primary pathology evaluation of the surgical specimen and contributed to the writing process. JGC: performed the clinical evaluation of the patient and contributed to the writing process. AM: coordinated and supervised the writing process, performed critical revisions of the text and figures, and is the corresponding author. All authors approved the final manuscript version.

## Consent for Publication

Informed and written consent were obtained from the patient.

## Conflict of Interest

The authors have no conflict of interests to disclose.
